# *Plasmodium vivax* Cell Traversal Protein for Ookinetes and Sporozoites (PvCelTOS) gene sequence and potential epitopes are highly conserved among isolates from different regions of Brazilian Amazon

**DOI:** 10.1371/journal.pntd.0005344

**Published:** 2017-02-03

**Authors:** Lana Bitencourt Chaves, Daiana de Souza Perce-da-Silva, Rodrigo Nunes Rodrigues-da-Silva, João Hermínio Martins da Silva, Gustavo Capatti Cassiano, Ricardo Luiz Dantas Machado, Lilian Rose Pratt-Riccio, Dalma Maria Banic, Josué da Costa Lima-Junior

**Affiliations:** 1 Laboratory of Immunoparasitology, Oswaldo Cruz Institute, Fiocruz, Rio de Janeiro, Rio de Janeiro, Brazil; 2 Laboratory of Clinical Immunology, Oswaldo Cruz Institute, Fiocruz, Rio de Janeiro, Rio de Janeiro, Brazil; 3 Computational Modeling Group - FIOCRUZ - CE, Fortaleza, Brazil; 4 Laboratory of Tropical Diseases - Prof. Luiz Jacintho da Silva, Department of Genetics, Evolution and Bioagents, University of Campinas, Campinas, São Paulo, Brazil; 5 Malaria Immunogenetic Laboratory, Instituto Evandro Chagas, Ananindeua, Pará, Brazil; 6 Laboratory of Malaria Research, Oswaldo Cruz Institute, Fiocruz, Rio de Janeiro, Rio de Janeiro, Brazil; Mahidol University, THAILAND

## Abstract

The *Plasmodium vivax* Cell-traversal protein for ookinetes and sporozoites (PvCelTOS) plays an important role in the traversal of host cells. Although essential to PvCelTOS progress as a vaccine candidate, its genetic diversity remains uncharted. Therefore, we investigated the PvCelTOS genetic polymorphism in 119 field isolates from five different regions of Brazilian Amazon (Manaus, Novo Repartimento, Porto Velho, Plácido de Castro and Oiapoque). Moreover, we also evaluated the potential impact of non-synonymous mutations found in the predicted structure and epitopes of PvCelTOS. The field isolates showed high similarity (99.3% of bp) with the reference Sal-1 strain, presenting only four Single-Nucleotide Polymorphisms (SNP) at positions 24A, 28A, 109A and 352C. The frequency of synonymous C109A (82%) was higher than all others (p<0.0001). However, the non-synonymous G28A and G352C were observed in 9.2% and 11.7% isolates. The great majority of the isolates (79.8%) revealed complete amino acid sequence homology with Sal-1, 10.9% presented complete homology with Brazil I and two undescribed PvCelTOS sequences were observed in 9.2% field isolates. Concerning the prediction analysis, the N-terminal substitution (Gly10Ser) was predicted to be within a B-cell epitope (PvCelTOS Accession Nos. AB194053.1) and exposed at the protein surface, while the Val118Leu substitution was not a predicted epitope. Therefore, our data suggest that although G28A SNP might interfere in potential B-cell epitopes at PvCelTOS N-terminal region the gene sequence is highly conserved among the isolates from different geographic regions, which is an important feature to be taken into account when evaluating its potential as a vaccine candidate.

## Author summary

Cell-traversal protein for ookinetes and sporozoites (CelTOS) presents a pivotal role in the cell traversal of host cells in mosquito and vertebrate hosts. For this reason, it has been considered a potential novel alternative for a vaccine against malaria caused by *P*. *falciparum*. However, little is known about its orthologous *P*. *vivax* CelTOS. Although the genetic diversity of this protein could be a limiting factor for acquisition of immunity and present implications for an effective vaccine development, it has never been explored. Thus, considering that the epidemiology of malaria in Brazil presents variable transmission rates and the knowledge on the genetic polymorphism of PvCelTOS remains unknown, we aimed to identify the *pvceltos* gene in isolates from five different regions of the Brazilian Amazon and to study the potential impacts of the genetic diversity of PvCelTOS in protein structures and predicted epitopes. Our findings indicate that PvCelTOS is an extremely conserved protein, presenting only four SNPs in the entire sequences of field isolates from Brazilian Amazon. The two non-synonymous mutations found in our field isolates presented no significant effect on the protein structure and a very low impact on potential T and B-cell epitopes indicated by our epitope prediction. Collectively, our data suggest that the small need to avoid the immune recognition by the human host and its importance on the parasite’s survival and transmission reflects a very conservative profile of *pvceltos* gene in field samples from Brazil and other endemic areas worldwide.

## Introduction

Malaria is an infectious parasitic disease with high prevalence and morbidity. Globally, it is estimated that 3.2 billion people in 95 countries and territories are at risk of being infected and develop the disease. In 2015, malaria caused an estimate of 438,000 deaths, mostly in African children [1]. Among the protozoa species causative of human malaria, *Plasmodium vivax*, although less prevalent than *P*. *falciparum* in absolute numbers, presents the world's largest spread, an increasing morbidity [2] and became the main cause of malaria outside Africa. In Brazil, although there are three species of *Plasmodium* that cause malaria (*P*. *falciparum*, *P*. *vivax* and *P*. *malariae*), approximately 87% of the 142,000 cases reported in 2015 were caused by *P*. *vivax* [3]. Thus, it is extremely important to develop new methods and intervention strategies to block or reduce this transmission.

Significant effort and progress on *P*. *vivax* control have occurred over the last years, but the understanding of *P*. *vivax* biology is still crucial to develop potential vaccines and to achieve the goal of eliminating malaria. The ability of the *Plasmodium* to recognize, and then invade hepatocytes or red blood cells, is central to the life cycle and also to the disease process. During the pre-erythrocytic stage, it is well established that *Plasmodium* sporozoites migrate through Kupffer cells and several hepatocytes before finally infecting a hepatocyte. Therefore, antigens located on the surface of the parasite or specifically in apical organelles of the parasite during this stage have been suggested as a target for a better understanding of *Plasmodium* lifecycle and, consequently possibly used as vaccine [4]. In this context, the Cell-Traversal protein for Ookinetes and Sporozoites (CelTOS) has been considered a new alternative for vaccine development [5,6]. This protein, secreted by micronemes, is important to the success of cell crossing by sporozoites and ookinetes, and also hepatocyte invasion carried out by sporozoites. Studies have shown that the disruption of the CelTOS gene encoding, in *P*. *berghei*, reduces the infectivity in the mosquito host and also the infectivity of the sporozoite in the liver, almost eliminating their ability to cell pass [7]. In addition, the CelTOS is necessary for the motility of the parasite in both the mosquito vector and the human host, being determinant for the success of malaria infections [8]. Recently, studies from Jimah et al. suggested that the CelTOS is responsible for breaking the cell membranes from the inside of infected human and mosquito cells to enable the parasites to exit and complete the traversal process (Jimah et al 2016). In relation to its potential as a vaccine candidate, antibodies against PfCelTOS were able to inhibit sporozoite traversal of hepatocytes [9], and induce protection in animals [10]. In humans, PfCelTOS derivative peptides elicited proliferative and IFN-γ responses in *ex vivo* ELISPOT assays using peripheral blood mononuclear cells (PBMCs) from irradiated sporozoite-immunized volunteers [8] and recombinant PfCelTOS were recognized by naturally acquired antibodies from exposed populations living in highly endemic areas from Africa [11]. However, all those previous studies used CelTOS protein of *P*. *falciparum* and/or *P*. *berghei*. Despite the antigenic and immunogenic properties of PfCelTOS, there is only one recent finding concerning the antigenic potential of its counterpart in *P*. *vivax*, the PvCelTOS, whose naturally acquired antibodies were able to recognize the recombinant protein [12].

Although essential to the development of its potential as a vaccine candidate, there is no available published data on the identification of *pvceltos* gene in field isolates and the evaluation of its genetic diversity in endemic areas. In fact, the extensive genetic diversity in natural parasite populations is a major obstacle for the development of an effective vaccine against the human malaria parasite, since antigenic diversity limits the efficacy of acquired protective immunity to malaria [13]. Despite the genetic diversity, which is one of the most prominent features of *P*. *vivax* infections, there is also a paucity of information on *celtos* gene polymorphism. Such data have importance in documenting the parasite genetic diversity changes and contribute to malaria control interventions in the future. Therefore, we proposed to identify *pvceltos* gene isolates from different regions of Brazilian Amazon and to study the potential impacts of the genetic diversity of PvCelTOS in protein structures and potential epitopes through bioinformatics tools.

## Methods

### Study sites and blood sample collection

**Fig 1:**
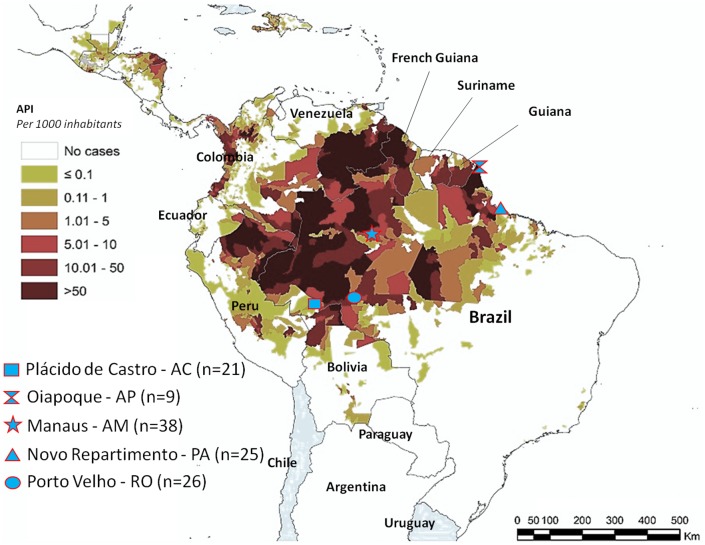
Geographical map showing the five study sites and the respective Annual Parasitic Incidence (API) (SIVEP-Malaria).

Most cases of malaria in Brazil are concentrated in the Amazon Region, an endemic area for the disease [14]. Therefore, the study was carried out in five different regions of Brazilian Amazon (Fig 1). A subset of 81 patients was analyzed out of 312 individuals previously evaluated by Cavasini *et al* (2007) [15] (21 individuals from Plácido de Castro, 9 individuals from Oiapoque, 25 individuals from Novo Repartimento and 26 individuals from Porto Velho) and, additionally, blood samples were collected from 38 *P*. *vivax* infected individuals from Manaus. Thus, a total of 119 blood samples were used in this study.

Plácido de Castro (PLC), is a city 90 km far from the capital of the State of Acre, located in Western Brazilian Amazon, with a population of 17,334 thousand inhabitants (16% aged above 18 years, at 153 meters above sea level, with a territorial area of 2,047,000 km^2^, latitude of -09° 58’ 29” and longitude of 67° 48’ 36”, where the main economic activities are cattle breeding, rubber agriculture and farming. Active malaria transmission takes place during all periods of the year.

Oiapoque (OIP), Amapá State, located in the Brazilian Eastern Amazon, a mining gold area, with 17,423 a thousand inhabitants, presenting latitude of 03° 49’ 58” and longitude of 51° 49’ 51”.

Manaus (MAO), the capital of Amazonas State, located in the Northern Region of Brazil, with a population of more than 2 million people. It is the most populous city of Amazonas state, presenting latitude of -03° 06’ 07” and longitude of -60° 01’ 3”.

Novo Repartimento (NR), is a city 600 km far from Belém, capital of the State of Pará, located in Brazilian Eastern Amazon, with 47,197 thousand inhabitants, at 460 meters above sea level, with a territorial area of 11,407 km^2^, presenting latitude of 04° 19’ 5” and longitude of 49° 47’ 47”, whose main economic activities are cattle breeding, commerce of manufactured products and farming. It presents active malaria transmission from January to December, with around 2,000 heterochthonous and autochthonous cases.

Porto Velho (PVL), capital of the Rondônia State, located in Western Brazilian Amazon, with a population of 360,068 thousand inhabitants (16% aged above 18 years), at 85 meters above sea level, with a territorial area of 34,082 km^2^, latitude of -08° 45’ 43” and longitude of 63° 45’ 43”, where the main economic activities are cattle breeding, rubber agriculture, wood exploration and farming. Active malaria transmission takes place during all periods of the year. The distances between the study sites are shown in Table 1.

**Table 1:** Distance in km between the five study sites.

	Distance (km)			
**Locality**	**OIP**	**MAO**	**NR**	**PVL**
**OIP**	-	-	-	-
**MAO**	1,196	-	-	-
**NR**	938	1,143	-	-
**PVL**	1,939	760	1,635	-
**PLC**	2,313	1,119	2,024	393

OIP: Oiapoque, AP; MAO: Manaus, AM; NR: Novo Repartimento, PA; PVL: Porto Velho, RO; PLC: Plácido de Castro, AC.

All *P*. *vivax* participants were enrolled according to the following criteria: sought medical assistance for clinical malaria symptoms, presented uncomplicated malaria symptoms, were > 18 years of age, and had a positive *P*. *vivax* malaria diagnosis. Pregnant women, patients < 18 years of age, and *P*. *vivax*- and *P*. *falciparum*-infected individuals were excluded from the study. Thin and thick blood smears were examined for the identification of the malaria parasite by a technician experienced in malaria diagnosis from the Brazilian Malaria Health Services. Thick blood smears from all of the subjects were stained with Giemsa, and a total of 200 microscopic fields were examined under a 1,000-fold magnification. Thin blood smears of the positive samples were examined for species identification. To increase the sensitivity of parasite detection, molecular analyses using specific primers for genus (*Plasmodium* sp) and species (*P*. *falciparum* and *P*. *vivax*) were performed in all of the samples as previously described. Donors positive for *P*. *vivax* and/or *P*. *falciparum* at the time of blood collection were subsequently treated by the chemotherapeutic regimen recommended by the Brazilian Ministry of Health.

### Ethical considerations

The study protocol was approved by the Research Ethics Committee of each locality, which included obtaining the following patients’ written consents for research use of their blood samples: Belém (Novo Repartimento/PA): 68473–970; Porto Velho (CEPEN): 76812–329; Rio Branco (Hospital Geral de Plácido de Castro/AC): 69928–000; Oiapoque (Hospital Municipal do Oiapoque/AP): 68980–000; Manaus (CEP-FIOCRUZ): 346–613. Written informed consents were obtained from all adult donors or from the parents of donors in the case of children. All the procedures adopted in this study fully complied with specific federal permits issued by the Brazilian Ministry of Health.

### Genomic DNA extraction

The DNA was extracted from blood samples using the QIAamp DNA blood midi kit (QIAgen) according to the manufacturer’s instructions and stored at -20°C until amplification.

### Design of PvCelTOS specific primers

The *pvceltos* gene is conserved among different species of *Plasmodium* and to obtain that of *P*. *vivax*, specific primers were designed using standard gene sequences of *P*. *vivax* Salvador-1 strain from NCBI database with Accession Nos. AB194053.1. All oligonucleotides were checked for specificity by using the Primer-BLAST tool provided by the National Center for Biotechnology Information (http://www.ncbi.nlm.nih.gov/tools/primer-blast/). The forward primer (5’-CCCCCAAAGGCAAAATGAACAA-3’) corresponded position 20 to 41 of the *pvceltos* gene sequence and the reverse primer (5’-AACTCATCTTCAGCTTCTTCCTC-3’) corresponded to position 569 to 547. The specific primers were chemically synthesized to perform PCR reaction and DNA sequencing.

### PCR amplification of *pvceltos* gene

The *pvceltos* gene was amplified in a conventional PCR method using the pair of primers PvCelTOS 5’—CCCCCAAAGGCAAAATGAACAA—3’ (forward) and PvCelTOS 5’—AACTCATCTTCAGCTTCTTCCTC—3’ (reverse). Amplification of the *pvceltos* gene was conducted in a reaction volume of 25 μL using 1 μL of DNA, 10 pmol/μL of each primer and the Master Mix kit (Promega) containing Taq DNA polymerase, PCR buffer and 10 nmol of each deoxynucleotide triphosphate (dNTP, Promega, Madison, WI USA). The conventional PCR reactions were carried out using a GeneAmp PCR system 9700 (Applied Biosystem) and the cycling conditions were as follows: one step at 95°C for 2 min.; 30 cycles at 95°C for 1 min., 57°C for 1 min. and 72°C for 1 min.; and a last step at 72°C for 1 min. In all reactions two negative controls were used (one without DNA and other with DNA extracted from *in vitro* culture of *P*. *falciparum* PSS1 strain) and a positive control (*P*. *vivax*-infected sample). To confirm the presence of DNA from the *in vitro* culture of *P*. *falciparum* and that the lack of amplification was due the specificity of the primers for PvCelTOS, we performed the amplification of the *P*. *falciparum* P126 gene fragment and electrophoresis as previously described [16]. Moreover, three *P*. *vivax*-infected samples from our study sites were randomly chosen. Five μL of PCR product were submitted to electrophoresis in 2% agarose gel (Sigma) in 1x TAE buffer (0.04 M TRIS-acetate, 1 mM EDTA) in the presence of 10x GelRed nucleic acid stain (Biotium) and afterwards the products were visualized by ultraviolet (UV) illumination. Sizing of products was performed using a GeneRuler 100 bp Plus DNA Ladder (Thermo Scientific). Then, PCR fragments were purified using the GE Healthcare Lifesciences kit according to the manufacturer’s protocol and sequenced.

### DNA sequencing and polymorphism analysis

The specificity of the assay was confirmed by sequencing the PCR products from all positive samples using a Big Dye terminator sequencing kit (Applied Biosystems) following the manufacturer’s instructions. The DNA sequencing was carried out on the 3730xl DNA analyzer (Applied Biosystems) and the results were analyzed using DNASTAR's sequence alignment software to identify polymorphism relative to the Sal-1 reference sequence from NCBI.

### 3D model and electrostatic analysis of PvCelTOS

The 3D structure of PvCelTOS was predicted using the Robetta algorithm [17]. The amino acid sequence was retrieved from NCBI under Accession Nos. AB194053.1. The Robetta is an automated algorithm for predictions of the 3D structure of proteins through *ab initio* and comparative modeling. The first step is the search for structural homologs using BLAST [18] or PSI-BLAST [19]. In the protein sequence, the target primary structure is broken down into separated domains, or independently folding units of proteins, by comparing the sequence to structural families in the Pfam database [20]. Domains with homolog structures follow a template-based modeling protocol. The final five structures are selected by taking the lowest energy models as determined by the Rosetta energy function. The electrostatic surface was calculated with the Adaptive Poisson-Boltzmann Solver (APBS) software [21] integrated with Pymol. The APBS software solves the Poisson-Boltzmann equation in order to describe electrostatic interactions between solute in aqueous solution. Continuous electrostatics plays a very important role in determining ligand-protein and protein-protein binding kinetics.

### Prediction of linear B-cell and T-cell epitopes

The prediction of linear B-cell epitopes was carried out using the program BepiPred [22]. This software takes a single sequence in FASTA format input and each amino acid receives a prediction score based on Hidden Markov Model profiles of known antigens and incorporates propensity scale methods based on hydrophilicity and secondary structure prediction. For each input sequence the server outputs a prediction score. The positions of the linear B-cell epitopes are predicted to be located at the residues with the highest scores. In order to consider a given region as a valid linear B-cell epitope for PvCelTOS, the cut-off value of 0.35 was used to warrant similar values of specificity (0.75) and sensitivity (0.49). Therefore, the epitope score represents the average of the scores of at least eight consecutive amino acids above the cut-off, and the sequences with higher mean values were chosen as potential linear epitopes.

The differential binding of T-cell epitopes spanning the full PvCelTOS sequence were made on 4/18/2016 using the IEDB analysis resource Consensus tool [23] which combines predictions from ANN aka NetMHC (3.4) [24,25], SMM [26] and Comblib [27]. Considering lengths of 9 mers, the prediction score of each length was evaluated against 26 of the most frequent HLA alleles (HLA-A*01:01; HLA-A*02:01; HLA-A*11:01; HLA-A*23:01; HLA-A*25:01; HLA-A*26:01; HLA-A*30:01; HLA-A*31:01; HLA-A*32:01; HLA-A*68:01; HLA-B*08:01; HLA-B*15:01; HLA-B*18:01; HLA-B*35:01; HLA-B*38:01; HLA-B*39:01; HLA-B*40:01; HLA-B*46:01; HLA-B*48:01; HLA-B*51:01; HLA-B*53:01; HLA-B*57:01; HLA-B*58:01; HLA-C*04:01; HLA-C*05:01; HLA-C*07:01). Peptides with median consensus percentile rank 20.0 as predicted binders and at least 60% of HLA binding frequency was considered potential T-cell epitopes.

### Statistical analysis

The one-sample Kolmogorov-Smirnoff test was used to determine whether a variable was normally distributed. Differences in proportions of haplotypes frequencies between studied localities were evaluated by the Fisher’s exact test using Prism 5.0 for Windows (GraphPad Software, Inc.). A two-sided P value < 0.05 was considered significant. Sequences were aligned using CLUSTAL X2 and the number of segregation sites (S), number of haplotypes, nucleotide diversity (π) and haplotype diversity were computed using DnaSP v5 [28]. The Tajima’s D test [29] for determining departure from the predictions of the neutral theory of evolution was also estimated with DnaSP v5. The genetic differentiation between populations was investigated evaluating the rate of fixation (F_ST_) by analysis of molecular variance (AMOVA) implemented in ARLEQUIN v3.5.2.2 [30] and significances were estimated using 10,000 permutations. The significance level was adjusted by Bonferroni correction for multiple tests.

## Results

### Standardization and molecular characterization of PvCelTOS in the studied regions

**Fig 2:**
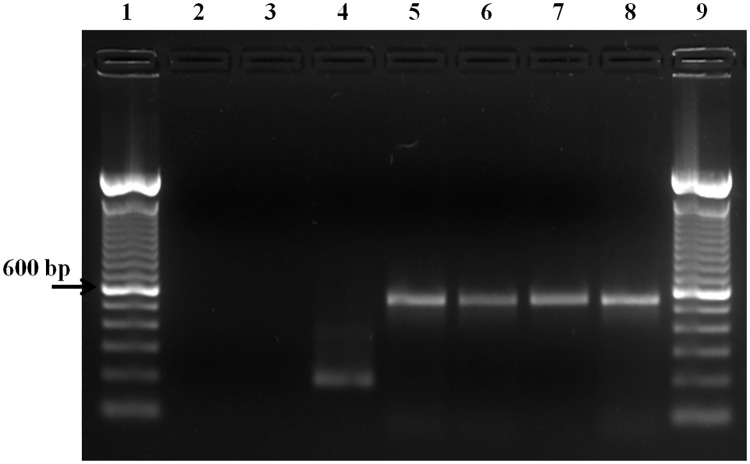
PCR amplification of the *pvceltos* gene. Fig 2 shows in **Lane 1**: 100 bp Molecular Marker; **Lane 2**: Negative control (water); **Lane 3**: *In vitro* culture of *P*. *falciparum* (amplification with PvCelTOS primers); **Lane 4**: *In vitro* culture of *P*. *falciparum* (amplification with p126 primers); **Lane 5**: PCR positive control (*P*. *vivax*-infected sample); **Lanes 6, 7 and 8**: samples; **Lane 9**: 100 bp Molecular Marker.

In order to identify the gene encoding the PvCelTOS in isolates from Brazilian endemic areas, 119 blood samples from infected individuals living in the cities of Porto Velho, Plácido de Castro, Manaus, Novo Repartimento and Oiapoque had the DNA extracted and subjected to molecular diagnosis by PCR. The primers designed from the Primer-BLAST program and PCR analysis by agarose gel revealed the amplification in 100% samples. All field isolates presented only one type of fragment corresponding to 550 base pair (bp). In addition to these samples, *P*. *falciparum* specimens were also tested, but proved negative for PCR amplification of the *pvceltos* gene (Fig 2). Therefore, the 119 samples from individuals infected with *P*. *vivax* amplified by PCR were subjected to sequencing reactions in order to screen the possible single nucleotide polymorphisms of the gene encoding the PvCelTOS.

### *Pvceltos* gene is highly conserved among field isolates, but presents few synonymous and non-synonymous mutations at specific positions

**Fig 3:**
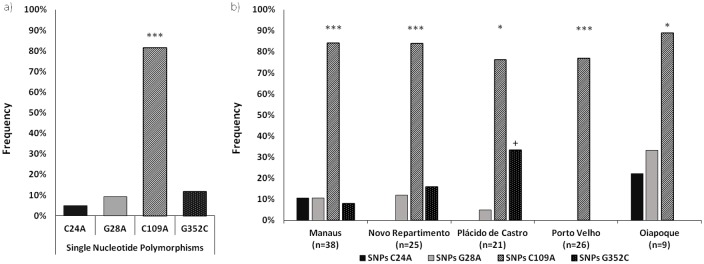
Analysis of genetic diversity of PvCelTOS in *Plasmodium vivax* isolates. (A) Fig A represents four mutations in specific bp positions (24, 28, 109 and 352). (B) The graphic represents the frequency of mutations in isolates from each studied locality. The black bar indicates the synonymous mutation C24A; the gray bar, the non-synonymous mutations G28A; the striped bar, the synonymous mutation C109A and the white dotted bar represents the non-synonymous G352C. (*) Indicates that the differences between the frequency of SNP C109A was higher than that of other mutations by exact test and (+) indicates that the frequency of SNP G352C was higher than the frequency of SNPs C24A and G28A by Fisher’s. (*): p<0.05; (**): p<0.01; (***): p<0.0001.

Standard gene sequences of *P*. *vivax* Salvador-1 (Sal-1) encoding PvCelTOS were aligned to sequences from different regions of Brazilian Amazon isolates. Identification of variants and novel haplotypes was done and our interpretations were confirmed with available standard gene sequence of the *P*. *vivax* CelTOS in PubMed database. The polymorphism identification in the gene encoding the PvCelTOS from our studied regions revealed that all isolates had a high degree of similarity in relation to base pair alignments with the reference strain (99.3%). However, from the 550 bp sequenced and aligned, four nucleotide bases (0.7%) presented mutations in specific bp positions (24, 28, 109 and 352), shown in Table 2. Interestingly, we did not detect point mutations in a single field or geographic area and all SNPs were present in at least two isolates and two sampling localities. Even with the high conservation degree of *pvceltos* gene sequence, 85% of the studied isolates presented at least one SNP in relation to the reference strain. As shown in Fig 3a, the synonymous mutation C109A was present in 82% field isolates and was significantly higher than all other 3 mutations (p<0.0001), while the other synonymous mutation C24A was the least frequent mutation. Two non-synonymous mutations, G28A and G352C, which represent the substitution of Glycine for Serine and Valine for Leucine, respectively, were also detected in frequencies of 9.2% and 11.7%, respectively. In addition, regarding the endemic areas studied, the higher frequency of C109A was maintained in all localities. Manaus presented the highest diversity, since we detected all four mutations among the 38 samples, while Porto Velho presented the lowest diversity, with only the synonymous mutation C109A. Lastly, in field isolates from Plácido de Castro, the non-synonymous SNP G352C was also significantly higher than C24A (p = 0.0086) and G28A (p = 0.0480), while in all other localities this predominance did not occur (Fig 3b).

**Table 2:** Mutations and corresponding amino acid substitutions in *pvceltos* gene.

**Sequences**	**Nucleotide position**			
	**22 to 24**	**28 to 30**	**109 to 111**	**352 to 354**
Wild Type	CCC	GGC	CGG	GTG
Mutants	- - A	A - -	A - -	C - -
	**Amino acid position**			
	**8**	**10**	**37**	**118**
Wild Type	Pro	Gly	Arg	Val
Mutants	-	Ser	-	Leu

Nucleotide and amino acid positions

### PvCelTOS haplotype frequency in field isolates

Only 18 isolates (15%) maintained their sequences identical to the reference strain in positions 24, 28, 109 and 352 (H1 = CGCG). Furthermore, the mutations resulted in nine different haplotypes (H2 = AGCG; H3 = CACG; H4 = CGAG; H5 = CAAG; H6 = CGAC; H7 = AGAG, H8 = CAAC; H9 = AAAG), whose frequencies are shown in Table 3. Among all field isolates studied the haplotype H4 presented the highest frequency and was significantly higher when compared to the reference H1 (p<0.0001). On the other hand H2 (p<0.0001), H3 (p = 0.0002), H5 (0.0328), H7 (p = 0.0028), H8 (p<0.0001) and H9 (p<0.0001) presented significantly lower frequencies when compared to H1. However, regarding these haplotypes obtained from human isolates from the Amazon regions, we could not determine a genetic structure based on the localities. Therefore, we observed that H1 and H4 were present in all studied localities while H2, H8 and H9 were detected in only a single locality (Manaus, Novo Repartimento and Oiapoque respectively). Even though the haplotypes could not be segregated according to their geographic origin, Manaus and Novo Repartimento presented the highest diversity of field isolates with six different haplotypes, while Porto Velho presented the lowest diversity, with only two haplotypes, which were common to all localities (H1 and H4). Interestingly, despite the difference in number of field isolates, Oiapoque presented a high diversity of *pvceltos* gene sequence with five haplotypes while only four different haplotypes were detected in Plácido de Castro (Table 3). Due to the very high similarity among sequences from different geographic origins and the consequent lack of phylogenetic signal, it was not possible to analyze the haplotypes in reliable clades.

**Table 3:** Distribution of PvCelTOS haplotypes among five studied localities of Brazilian Amazon.

	Haplotypes PvCelTOS—n (%) (Nucleotide positions 24, 28, 109 and 352—CGCG)								
Locality	Ref. strain—H1 (CGCG)	H2 (AGCG)	H3 (CACG)	H4 (CGAG)	H5 (CAAG)	H6 (CGAC)	H7 (AGAG)	H8 (CAAC)	H9 (AAAG)
Manaus (n = 38)	**5 (13.1%)**	1 (2.6%)	-	22 (57.8%) ***	4 (10.5%)	3 (7.9%)	3 (7.9%)	-	-
Novo Repartimento (n = 25)	**3 (12%)**	-	1 (4%)	16 (64%) ***	1 (4%)	3 (12%)	-	1 (4%)	-
Plácido de Castro (n = 21)	**3 (14.3%)**	-	1 (4.8%)	10 (47.6%)*	-	7 (33.3%)	-	-	-
Porto Velho (n = 26)	**6 (23.1%)**	-	-	20 (76.9%) ***	-	-	-	-	-
Oiapoque (n = 9)	**1 (11.1%)**	-	-	4 (44.4%)	2 (22.2%)	-	1 (11.1%)	-	1 (11.1%)
**Total (n = 119)**	**18 (15.1%)**	1 (0.8%)***	2 (1.7%) ***	72 (60.5%) ***	7 (5.9%) *	13 (10.9%)	4 (3.4%) **	1 (0.8%) ***	1 (0.8%) ***

The values represent the number and frequency (%) of found haplotypes on each studied locality. H4 represents the consensus sequence. (*) Indicates that the difference between the frequencies of mutate haplotype and reference strain (H1) was significant by Fisher’s exact test.

(*): p<0.05;

(**): p<0.01;

(***): p<0.0001.

### Population genetic analysis

We sequenced *pvceltos* gene (positions 19–569) of 119 samples collected from five regions of Brazilian Amazon. From the alignment with reference strain (Sal-1), four distinct SNPs were identified. Two SNPs were synonymous (C24A and 109A) and two were non-synonymous (G28A and G352C). The nucleotide diversity (π) for *pvceltos* of 119 sequences analyzed was 0.00141 ± 0.00014. The highest nucleotide diversity was observed in the Oiapoque group (0.00202 ± 0.00044), followed by the Plácido de Castro group (0.00161 ± 0.00029). Among all 5 populations, Porto Velho sequences displayed the lowest nucleotide diversity (0.00067 ± 0.00017) as expected, since only one SNP was detected in this group (Table 4). Similarly, parasites from Oiapoque presented the highest estimate of haplotype diversity (*H*_*d*_*)* (0.806 ± 0.014) whereas parasites from Porto Velho showed the lowest *H*_*d*_ (0.369 ± 0.091). Haplotype diversity was similar among the other studied areas (Table 4). The Tajima’s D test was performed to asses if there is selective pressure on the *pvceltos* gene. Although the Tajima’s D values ranged between -0.279 and 0.699, tests showed no significant departures from neutrality in all studied areas, indicating no significant selection in the *pvceltos* gene (Table 4).

**Table 4:** Comparison of genetic diversity among isolates from Brazil.

	No. of segregating sites (S)	No. of haplotypes	Haplotype diversity (*H*_*d*_)	Nucleotide diversity (π)	Tajima’s test
**Novo Repartimento**	3	6	0.58	0.00142	-0.045^ns^
**Manaus**	4	6	0.66	0.00153	-0.279^ns^
**Oiapoque**	3	5	0.81	0.00202	0.025^ns^
**Porto Velho**	1	2	0.37	0.00067	0.699^ns^
**Plácido de Castro**	3	4	0.67	0.00161	0.163^ns^
**All samples**	4	9	0.61	0.00141	0.077^ns^

The extent of *pvceltos* gene sequence corresponds to nucleotides 19–569 (reference clone Sal-1).

^ns^: not significant (p > 0.10).

Pairwise comparisons between each parasite population were performed using the F_ST_ statistics to check whether there was indicative of genetic differentiation between parasite populations, but all F_ST_ values were non-significant, suggesting lack of genetic differentiation between the studied populations (Table 5).

**Table 5:** Genetic differentiation between samples from Brazil, measured by pairwise *F*_ST_ values.

	**Novo Repartimento**	**Manaus**	**Oiapoque**	**Porto Velho**
**Novo Repartimento**	-	-	-	-
**Manaus**	-0.015	-	-	-
**Oiapoque**	0.056	0.010	-	-
**Porto Velho**	0.036	0.015	0.196	-
**Plácido de Castro**	-0.004	0.048	0.143	0.123

The *F*_ST_ values are not significant after Bonferroni correction (p > 0.05).

### Non-synonymous mutations reveal low diversity of PvCelTOS protein sequence in relation to genome sequences available worldwide

**Fig 4:**
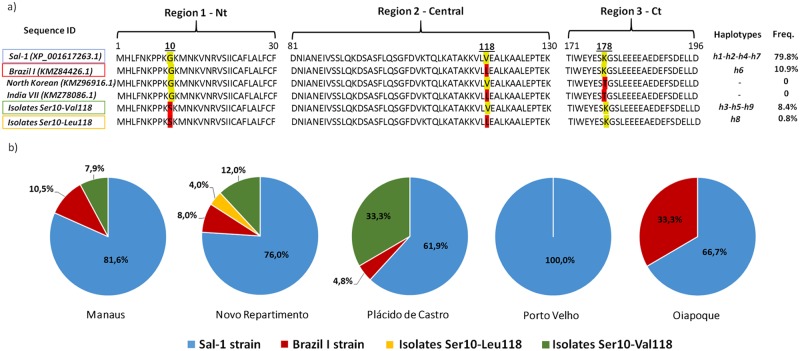
Alignment of protein sequences and frequency of mutations on field isolates. (A) CelTOS protein derivatives from *P*. *vivax* genome. The yellow mers represent the reference strain and the red mers the described mutate amino acid. (B) Frequencies of mutations in isolates from different Brazilian Amazon regions, where the colors blue, red, yellow and green represent genome identical to strains Sal-1; Brazil I and mutant isolates Ser10-Leu118 and Ser10-Val118, respectively.

The detected non-synonymous mutations characterized the specific amino acid substitutions in positions 10 (Glycine for Serine) and 118 (Valine for Leucine). As observed in the protein sequence alignments, PvCelTOS also presented high amino acid sequence conservation degree, since only 24 isolates (19.2%) presented non-synonymous mutations and had different sequences in comparison with the reference Sal-1 strain, whose frequency was significantly higher than all other protein sequences found in our field isolates (79.8%; p<0.0001). Therefore, we subsequently aligned the protein sequence of these mutant field isolates in relation to other three hypothetical CelTOS protein derivatives from *P*. *vivax* genome data available in PubMed protein database (Fig 4a). Only 13 isolates (10.9%) presented sequences identical to Brazil I strain and none of our field isolates presented complete homology with North Korean and India VII strains, however both Asian strains also presented mutations in C terminal region at position 178 (Lysine for Threonine) that was not detected in our Amazon isolates. Interestingly, the N-terminal mutation at position 10 (Gly10Ser) was never detected in available sequences, but it was present in 9.2% of our field samples. Regarding the five regions studied, all isolates from Porto Velho presented full homology with Sal-1 amino acid sequence, while in other regions the frequencies of mutant sequences ranged from 21% to 44% (Fig 4b). Noting the diversity identified following the *pvceltos* gene, our data indicate that it is limited in isolates from different regions of the Brazilian Amazon. However, these two non-synonymous mutations found may have an impact on the protein folding and also influence its potential as an epitope.

### Non-synonymous mutations could modify the potential of predicted B-cell epitopes but not for T_CD8+_ epitopes.

**Fig 5:**
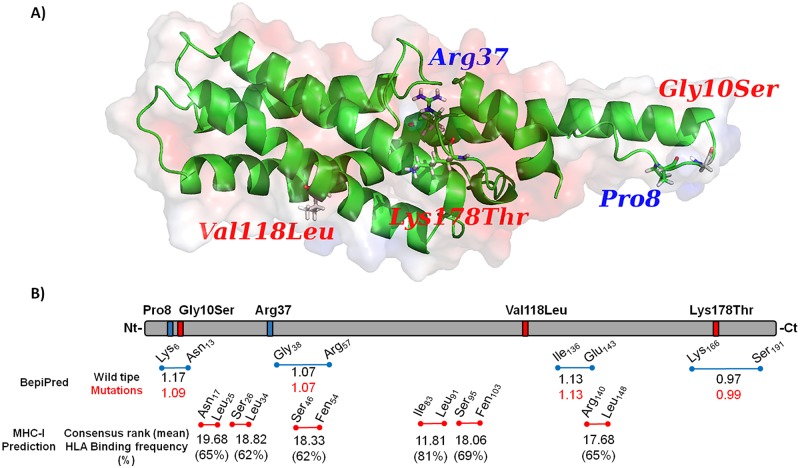
Modeling structure and *In silico* analysis of PvCelTOS. (A) Synonymous and non-synonymous mutations were illustrated by blue title and red title, respectively, on 3D structure of PvCelTOS. The red and blue clouds represent the negative and positive surface, respectively. (B) Synonymous and non-synonymous mutations found in our population and other described mutations are illustrated by blue bars and red bars, respectively, on PvCelTOS structure. The blue lines represent predicted linear B-cell epitopes and the red lines represent predicted T_CD8+_ epitopes. On both (B and T predicted epitopes) the letter and number of each epitope indicate the C-terminal and N-terminal amino acid. The BepiPred values represent the predicted score of linear B-cell epitope in wild type haplotype (H1) and mutate strain (red number). The IEDB MHC-I indicates the mean binding prediction score of T_CD8+_ epitopes and respective HLA binding frequency among 27 evaluated HLA. No differences of prediction T-cell epitopes are observed between wild types or mutate PvCelTOS.

Fig 5a depicts the electrostatic potential around the mutations. The region encompassing Arg37 shows a strong negatively charged surface. The Lys178 position showed the same negative pattern, while Val118 and Gly10 are positively charged. Pro8 region is mostly neutral. Arg37 and Val118 are part of a stable alpha helix structure, whereas Pro8, Gly10, and Lys178 belong to flexible loop structures. Also, all residues are exposed to the surface, except Arg37 which is hidden inside the negative pocket. As shown in Fig 5b four high scored potential linear epitopes with at least eight amino acids were identified in the entire protein sequence (Lys6-Asn13; Gly38-Arg57; Ile136-Glu143 and Lys166-Ser191). The prediction scores ranged from 0.97 to 1.17 and no immunodominant epitopes could be identified by this approach. Considering that two of non-synonymous mutations were inserted in predicted B-cell linear epitopes (Gly10Ser and Lys178Thr), we analyzed the prediction scores of mutate epitopes. Interestingly, the C-terminal mutation Lys178Thr, observed only in Asian strains, North Korean (KMZ96916.1) and Indian VII (KMZ78086.1), resulted in a slight increase of the predicted score; while the N-terminal mutation Gly10Ser, observed in our Brazilian isolates Ser10-Val118 and Ser10-Leu118, resulted in a decrease of the predicted score for an epitope (Fig 5b). On the other hand, the predicted T_CD8+_ epitopes were conserved among all known strains and isolates, once non-synonymous mutations were not observed inside these epitopes. Analyzing the full sequence of PvCelTOS, six T_CD8+_ predicted epitopes presented consensus score smaller than 20 and were predicted to be recognized by more than 60% of analyzed HLA (Fig 5b). Among these epitopes, the sequence RVSEDAYFL (PvCelTOS_I83-L91_) was considered a potential promiscuous T_CD8+_ epitope, since it was predicted as bonded by 81% of evaluated HLAs and presented a mean consensus score of 11.81. However, the potential of predicted epitopes as target of immune response and the effects of mutations on immune response against PvCelTOS remain unexplored.

## Discussion

Cell-traversal protein for ookinetes and sporozoites (CelTOS) has been considered a potential novel alternative for a vaccine against malaria. Although the biological function is not completely elucidated, its pivotal role in the cell traversal of host cells in mosquito and vertebrate host is important to a successful hepatocyte traversal and infection. Immunologic studies have demonstrated that CelTOS is target of naturally acquired cellular [8] and humoral response in exposed individuals [9]. However, one of the major obstacles to malaria vaccine development is still the low efficiency in inducing protection, which, in part, can be explained by genetic polymorphisms encoding different proteins used as immunogens [31]. In this context, the genome sequence of various organisms and the advances in bioinformatics have revolutionized the field of vaccinology, allowing the identification of vaccine candidates presenting low antigenic variation. Actually, several studies concerning the genetic diversity of *Plasmodium* spp. have described *P*. *vivax* and the gene coding for antigenic determinants such as circumsporozoite surface protein (CSP) [32], Merozoite Surface Proteins (MSP) [33], Duffy Binding Protein (DBP) [34] and Apical Membrane Antigen-1 (AMA-1) [35]. (Reviewed by [36]). In fact, the genetic diversity of these proteins in hyperendemic areas has been described as a limiting factor for the rapid acquisition of protective immunity, and as a consequence for the development of an effective vaccine. Moreover, the antigenic polymorphism of *P*. *vivax* vaccine candidates has been little discussed in unstable transmission areas such as the Brazilian endemic areas. Thus, considering that the epidemiology of malaria in Brazil presents unstable transmission and the knowledge about the genetic polymorphism of PvCelTOS remains unknown, we aimed to identify the *pvceltos* gene in isolates from different regions of the Brazilian Amazon and to study the potential impacts of the genetic diversity of PvCelTOS in protein structures and predicted epitopes.

The identification and evaluation of the genetic diversity of *pvceltos* gene in isolates from different geographic regions has not been previously studied and this was the first report. Despite the large distance among the studied localities and the possible existence of a gene flow of *Plasmodium vivax* genome among the studied populations which, associated with migration of people, could promote the gene flow of the parasite [37], our first results showed that *pvceltos* gene is highly conserved, presenting only 4 SNPs along its entire sequence, 2 synonymous and 2 non-synonymous mutations. This high conservation degree was expected, once it has been shown that CelTOS amino acid sequence is partially conserved even among three different *Plasmodium* species (*P*. *vivax*, *P*. *falciparum* and *P*. *berghei*) [7]. In relation to specific *P*. *vivax celtos* gene, there is a paucity of information available. In fact, it was described for only four different strains used in complete genome studies: Sal-1, Brazil I, North Korean and India VII. Therefore, even with the high conservation degree of *pvceltos* gene sequence in relation to the reference strain Sal-1, all these strains also presented at least one SNP. In our studied isolates, the synonymous mutation C109A was predominant and significantly higher than all other 3 mutations found, while the other synonymous mutation C24A was the least frequent mutation. It is important to mention that this predominant mutation (C109A) is also present in human P01 strain, a new reference genome for *P*. *vivax* from an Indonesian clinical isolate [38]. Classically, synonymous changes were thought to have no effect on the protein and were called silent, however, recent studies show that even synonymous nucleotide changes can affect protein folding and function [39–41] (Reviewed by [42]). Indeed, in most of the gene encoding proteins, the rate of synonymous substitutions is higher than the rate of non-synonymous substitutions, a condition known as purifying selection, and this has been demonstrated in other *Plasmodium* proteins, such as PfAMA-1 [43]. Interestingly, in relation to *pvceltos* we observed a perfect balance of synonymous and non-synonymous substitutions in the few polymorphisms found in all gene sequences among geographically distinct isolates.

This balance and the low diversity observed could raise at least two hypothesis: firstly, a possible low selective pressure of the immune system against this antigen, which can be corroborated by recent findings from Longley and colleagues that demonstrated a low frequency of naturally acquired antibodies against PvCelTOS in comparison with other sporozoite antigens such as CSP [44]; secondly, the high importance of this protein in sporozoite and ookinetes traversal process could be a consequence of this high conserved profile observed in the sequences of our study. Therefore, aiming to evaluate the degree of diversity of PvCelTOS in different field isolates from Brazilian Amazon, we also compared the amino acid sequence of each field isolate with the reference strain (Sal-1) and the three other hypothetical CelTOS protein derivatives from *P*. *vivax* genome (Brazil I, North Korean and India VII). Curiously, our isolates presented higher similarity in relation to the reference strain than to Brazil I strain which presented identical sequences in only 13 isolates. Additionally, none of our field isolates presented complete homology with North Korean and India VII strains, both Asian strains presented a mutation in C terminal region at position 178 that was not detected in our Amazon isolates. Moreover, we observed an N-terminal mutation at position 10 (Gly10Ser), which had never been detected in available sequences, but was present in 9.2% of our field samples, as isolates Ser10-Leu118 and Ser10-Val118. This mutation was present in three distant study localities (Manaus, Novo Repartimento and Plácido de Castro) and it was more frequent than the sequence of Brazil I strain in Novo Repartimento and Plácido de Castro. Interestingly, although the distance from Novo Repartimento, Plácido de Castro and Manaus to Oiapoque could difficult the gene flow and thus explain the absence of this mutation in Oiapoque population, the low frequency of gene flow promoted by the distance would not be the reason for the absence of this mutation in populations; since Porto Velho, which is closer to Plácido Castro (the locality with the highest frequency of this mutation), did not present this mutation.

Unfortunately, due to this high similarity degree we could not determine a genetic structure based on the localities, and the sequences and haplotypes could not be eligible to construct a phylogenetic tree. However, it was possible to identify 9 different haplotypes of *pvceltos* among the 119 *P*. *vivax* field isolates from the Amazon regions that were analyzed. Regarding the *pvceltos* sequences, we observed that haplotype H1 and H4 were present in all studied localities, however haplotype H4 presented the highest frequency and was significantly higher when compared to the reference H1. These findings suggest a global distribution of parasites containing similar *pvceltos* genotypes. Moreover, the existence of the same haplotypes in different malaria endemic areas will be important for the rationale of malaria vaccine designs.

Like other antigens of pre-erythrocyte stage, the immunity focused on CelTOS depends on humoral and cellular immune responses [10]. Antibodies induced by immunization with *P*. *berghei* CelTOS were able to recognize live as well as fixed *P*. *berghei* sporozoites [10] and immunization with *P*. *falciparum* CelTOS elicits cross-species protection against heterologous challenge with *P*. *berghei* [9]. Despite this cross-species reactivity, the low degree of similarity between the *P*. *falciparum* and *P*. *vivax* CelTOS (63%), and the knowledge that the protection can be reduced by depleting T-cell subsets in immunized animals prior to the sporozoite challenge thus eliminating the contribution of cellular components in protection [10], make crucial the evaluation of both arms of the adaptive response against PvCelTOS to validate it as a vaccine candidate. Additionally, studies based on the genetic diversity of *P*. *falciparum* merozoite surface proteins, have demonstrated that non-synonymous SNPs contribute to the variability of the parasite and provide escape from host immunity [45]. Thus, to assess the targets of immune response in PvCelTOS and evaluate the potential effects of non-synonymous mutations on immune response against PvCelTOS, we used *in silico* approaches to determine differences on predicted T_CD8+_ epitopes and linear B-cell epitopes among the reference strain (Salvador-1) and mutant PvCelTOS. Firstly, four epitopes were predicted as linear B-cell epitopes on full sequence of PvCelTOS. Interestingly, non-synonymous mutations could modify the potential of these predicted epitopes, once the N-terminal and C-terminal described non-synonymous mutations (Gly10Ser and Lys178Thr) were inserted in predicted linear B-cell epitopes and affected its prediction score. We hypothesize that this finding could not justify the low frequency of responders observed in the unique work that evaluated the natural immune response against PvCelTOS on exposed individuals from Western Thailand [44], but it could indicate the genetic diversity of *Plasmodium vivax* and therefore, its possible effects on immune response can be considered in future studies. Moreover, it has been demonstrated that few amino acid changes can prejudice the binding of peptides to MHC molecules, reduce recognition by T cells or generate antagonistic peptides that inhibit activation of specific T cells by the MHC-peptide complex (Reviewed by [42]). Therefore, in relation to potential T-cell epitopes, six T_CD8+_ epitopes were predicted as hypothetical promiscuous epitopes, presenting an HLA binding frequency higher than 60% and a mean consensus rank smaller than 20. Curiously, PvCelTOS has conserved T_CD8+_ epitopes among all different strains and isolates; once there are not non-synonymous mutations inserted on any predicted T-cell epitope. This finding allied to the showed cellular response to *Plasmodium falciparum* CelTOS in exposed individuals [8] supports the necessity to identify and validate PvCelTOS T-cell epitopes that could be interesting on new vaccine approaches.

*P*. *vivax* displays almost twice as much genetic diversity as *P*. *falciparum* in terms of SNP diversity and gene family variability. This implies that the global population of *P*. *vivax* may have a capacity for greater functional variation, mainly in gene families associated with immune evasion and erythrocyte invasion. In summary, our findings in PvCelTOS indicate that the very low variations in gene sequences could suggest that this conservative profile is important to the parasite’s survival and transmission. Moreover, although some studies have shown the influence of positive natural selection on genetic variability of other *P*. *vivax* vaccine candidates such as PvAMA-1, PvDBP and PvTRAP [46–48], our epitope prediction results indicate that the few CelTOS polymorphism in *P*. *vivax* is not maintained by balancing selection related to avoidance of immune recognition by the human host. However, future investigations aiming the naturally acquired cellular and humoral immune response against PvCelTOS derived antigens are still needed to corroborate the potential of PvCelTOS as a vaccine candidate.

## Genes and protein sequences used

*Plasmodium vivax* pvCelTOS mRNA for Pv cell-traversal protein, complete CDs. Accession number: AB194053.1; S4 [*Plasmodium vivax* Sal-1] Accession number: XP_001617263.1; Hypothetical protein PVBG_00206 [Plasmodium vivax Brazil I] Accession number: KMZ84426.1; Hypothetical protein PVNG_01740 [Plasmodium vivax North Korean] Accession number: KMZ 96916.1; Hypothetical protein PVIIG_00773 [Plasmodium vivax India VII] Accession number: KMZ 78086.1
